# Emerging technologies for single-cell glycomics

**DOI:** 10.1016/j.bbadva.2024.100125

**Published:** 2024-11-26

**Authors:** Sunada Keisham, Hiroaki Tateno

**Affiliations:** a Cellular and Molecular Biotechnology Research Institute, Multicellular System Regulation Research Group, National Institute of Advanced Industrial Science and Technology (AIST), Central 6, 1-1-1 Higashi, Tsukuba, Ibaraki 305-8566, Japan; b Ph.D. Program in Human Biology, School of Integrative and Global Majors, University of Tsukuba, Tsukuba, Japan

**Keywords:** Glycan, Single-cell, Sequencing, Glycome, scGR-seq, Glycomics

## Abstract

Glycans are present on virtually all cellular surfaces and are important regulators of multicellular communications. Advances in single-cell omics technologies have revolutionized life science research by elucidating cellular heterogeneity through integrated multimodal analyses, providing a comprehensive view of cellular functions. However, dissecting the heterogeneity of glycans at the single-cell level has been challenging due to their structural complexity and unamplifiable nature. Recently, we developed a novel technology called single-cell glycan and RNA sequencing (scGR-seq), which converts glycan information into genetic information using DNA-barcoded lectins, amplifies it by PCR, and simultaneously measures the glycome and transcriptome in thousands of single cells on a next-generation sequencer. In this mini-review, we review the recent advances in single-cell glycomics, focusing on our scGR-seq technology.

## Highlights

• Recent advances in single-cell glycomics are outlined.
• The concept of glycan profiling by sequencing is summarized.
• The single-cell glycan and RNA sequencing (scGR-seq) method is reviewed.

## 1 Introduction

The outermost surface of all living cells is decorated with thick layers of carbohydrates, referred to as glycans. Glycans are essential building blocks of life, consisting of monosaccharides linked by glycosidic bonds [1], which are primarily modified on lipids or proteins, forming glycoconjugates such as glycoproteins, glycolipids, and proteoglycans [2]. Glycans undergo further modification by sulfation, acetylation, etc., and have branched structures and numerous isomers (anomeric and linkage isomers). This results in an extremely complex and diverse glycome. Glycans are secondary products of genes, generated by hundreds of competing and sequentially acting enzymes such as glycosyltransferases, glycosidases, epimerases, and sulfotransferases. In addition, glycosylation is influenced by various factors such as localization of enzymes, abundance of donor substrates, accessibility of glycans, residence time, competition between enzymes, and expression of chaperones, etc. [3,4]. Therefore, it is necessary to develop technologies that can directly analyze cell surface glycans.

## 2 Reading the glycome by lectins

Glycome, the total set of glycan expressed on cells or tissues, not only differs among cell types but also changes in response to the intracellular and extracellular environments. Therefore, glycans are closely linked to cellular states such as differentiation, tumorigenesis, aging, immune response, and viral infection [[5], [6], [7], [8]]. Various methods have been developed to analyze glycomics. These techniques include mass spectrometry (MS), high-performance liquid chromatography (HPLC), nuclear magnetic resonance (NMR), and capillary electrophoresis (CE) [[9], [10], [11]]. MS has been widely used because it enables detailed structural analysis of glycans, but it requires complicated pretreatment such as the release, separation, and purification of glycans from the aglycone. In 2005, lectin microarrays (LMA) were introduced as a technology for simple and rapid glycan profiling [12]. Lectin is a general term for a group of proteins that bind to glycans. Lectins are found in almost all organisms, from plants to humans, and have long been used as probes to read glyco-code because of their specificity for various glycan structures [13]. In lectin microarrays, ∼100 lectins with such glycan-binding specificity are spotted on glass slides and immobilized. Biological samples containing glycoproteins are fluorescently labeled and incubated with the lectin microarray. By measuring the amount of fluorescence on the spot of each lectin, the binding profile of each lectin to the glycans in the biological sample can be obtained. While LMA does not determine the exact glycan structure, it allows the extraction of the main characteristics of glycans, such as the type and amount ratio of glycan epitopes, degree of branching, degree of modification, and comparative analysis among the measured samples [14]. LMA can analyze biological samples without separating glycans from the aglycone, as long as the samples are fluorescently labeled. Various biological samples have been analyzed using LMA, including body fluids such as serum, plasma, and urine, as well as cell and tissue lysates, bacteria, whole cells, and exosomes [[15], [16], [17], [18], [19], [20]]. Reading the glycome by lectins, decoder molecules of the glyco-code in nature, is an alternative approach to gaining insights into glycans' biological functions from various biological samples.

## 3 Concept of glycan profiling by sequencing

Tissues and organs contain various cell types and cellular states determined by both cell-intrinsic and cell-extrinsic factors. Therefore, single-cell sequencing is indispensable for acquiring omics information about individual cells. In recent years, the rapid development of next-generation sequencing (NGS) at single-cell resolution has revolutionized molecular biology by uncovering the complex heterogeneity within cellular populations [21]. It is becoming possible to comprehensively characterize the state of the cell and its activities in both health and disease by simultaneously acquiring multiple omics information, including genomics, transcriptomics, proteomics, epigenomics, and metabolomics [22]. However, none of the conventional technologies can analyze the glycome at the single-cell level. Why is it so difficult to analyze glycan expression in single cells? One reason is that, unlike DNA and RNA, glycans cannot be amplified by PCR. Therefore, we hypothesized that conjugating lectins with an oligonucleotide sequence (DNA-barcoded lectin) would enable the transformation of glycan information to gene information, which can be amplified and subsequently measured using a next-generation sequencer. In addition, the simultaneous analysis of the glycome with other molecular profiles, such as the transcriptome, may be realized [23].

## 4 Plate-based scGR-seq

In 2016, we started developing a technology to analyze glycans in single cells as part of a Japan Science and Technology Agency (JST) PRESTO project. We conjugated lectins with known specificity to DNA oligonucleotides containing a barcode sequence unique to each lectin, allowing us to identify the specific lectins through DNA sequencing. The lectins were conjugated via their amino groups to the DNA oligonucleotides using a photocleavable dibenzocyclooctyne-N-hydroxysuccinimidyl ester (DBCO-NHS), which enabled efficient conjugation with 5′-azide-modified oligonucleotides through copper-free click chemistry. The oligonucleotides were then released from the lectin upon exposure to ultraviolet (UV) light [23]. We prepared 39 DNA-barcoded lectins specific to various glycan structures modified on glycoconjugates such as sialic acid, fucose, galactose, N-acetylglucosamine, and mannose. These DNA-barcoded lectins were used to react with cells, after which the single cells were separated into a PCR tube and exposed to UV light. Subsequently, the supernatants containing the released DNA barcodes were recovered, amplified by PCR, and analyzed by next-generation sequencing to quantify the DNA barcodes [23]. This method was named as Glycan-seq. The remaining cells were further processed for scRNA-seq (single-cell RNA sequencing) using a plate-based method called RamDA-seq, a full-length single-cell total RNA-sequencing method. The combined single-cell glycan and RNA sequencing we developed was named scGR-seq [23].

We first performed glycan profiling on bulk samples (1 × 10^5^ cell population) of various cell types, including fibroblasts, iPSCs, CHO glycosylation-defective mutant cells, and neural progenitor cells (NPCs), to evaluate the lectin binding profile obtained by Glycan-seq. The glycan profiles obtained by Glycan-seq were in good agreement with the results obtained by flow cytometry, a standard method for cell analysis [23]. scGR-seq was then applied to two different cell types: induced pluripotent stem cells (iPSCs) and NPCs after differentiation from iPSCs for 7 days, for the simultaneous analysis of RNA and glycan in single cells. Clustering the two cell types using Uniform Manifold Approximation and Projection (UMAP) based solely on RNA or glycan information did not achieve accurate classification. However, integrating RNA and glycan information accurately classified the cells into two distinct clusters, demonstrating improved accuracy in cell type classification. To evaluate the clustering by UMAP, the Adjusted Rand Index (ARI), a statistical analysis method for assessing clustering accuracy, was used to determine whether each cell could be correctly classified into two groups, iPSCs and NPCs, based on their molecular profiles. The ARIs of iPSCs and NPCs were 0.8 and 0.68, respectively, where a perfect match represents a value of 1. On the other hand, when cell classification was performed using data integrating both molecular profiles, the ARI was 1, indicating that the cells were correctly identified. This indicates that cell types can be more accurately classified by combining information from both RNA and glycans. Furthermore, when lectins with high reactivity to each cell type were extracted, the α1–2fucose-binding lectins, rBC2LCN and TJAII, showed the highest reactivity to iPSCs. The results aligned well with those previously obtained from lectin array analysis [5]. Additionally, rBC2LCN demonstrated the strongest correlation with the expression variation of *POU5F1*, a gene marker for pluripotent stem cells. This finding aligns with previous reports that rBC2LCN specifically binds to pluripotent stem cells, suggesting that glycans and genes are accurately mapped at the single-cell level [23].

Genes are annotated with information on related cellular functions in databases such as Gene Ontology (GO) and KEGG. Therefore, by analyzing the cellular functions of RNA groups (genes) that are highly correlated with glycans, it is possible to gain insights into the relationship between glycans and cellular functions. We classified highly correlated lectins and RNAs by partial least squares (PLS) regression [24] and found that fucose-binding lectins (rAAL), mannose-binding lectins (rBC2LA, rBanana), and galactose-binding lectins (rLSLN) are highly correlated with RNAs related to brain development and neuronal differentiation. These lectins showed increased reactivity during the differentiation process from iPSCs to NPCs, which is consistent with the previous findings that complex-type glycans are less abundant in neurons, while high mannose-type and hybrid-type N-glycans are more abundant [25,26]. Thus, scGR-seq can be used to investigate the association of glycan and gene expression [23].

## 5 Droplet-based scGR-seq

**Fig. 1:**
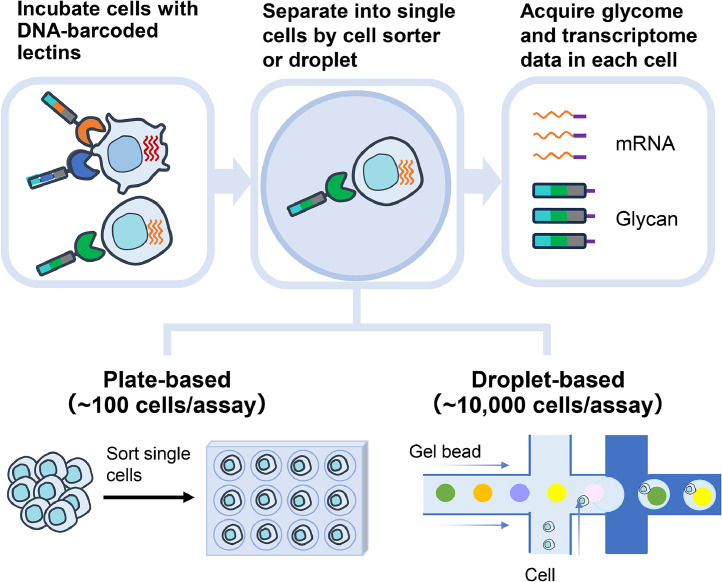
Schematic workflow of plate-based and droplet-based single-cell Glycan and RNA sequencing (scGR-seq).

Plate-based scGR-seq enables integrated analysis of glycan and RNA at the single-cell level. However, the sample size is limited to only a few hundred cells, as each cell must be manually processed for the single-cell analysis. Therefore, we adopted droplet microfluidic technology to scGR-seq and developed droplet-based scGR-seq, which enables the analysis of glycome and transcriptome in 10,000 single-cells at once. In droplet-based scGR-seq, 1 × 10^5^ cells are reacted with DNA-barcoded lectins. The unbound DNA-barcoded lectins were washed, followed by encapsulation of lectin-bound single cells with one gel bead to generate GEMs (gel beads in emulsion) using a microchannel. The beads contain different DNA barcodes serving as cell tags for each cell. Within each emulsion, the mRNA is reverse transcribed, and the DNA-barcoded lectins are simultaneously amplified along with the DNA barcodes from the beads. Thus, the mRNA and DNA barcodes of each lectin from the same cell are linked by the same cell tag, allowing for the simultaneous acquisition of transcriptome and glycome data for many individual cells [27] (Fig. 1).

Droplet-based scGR-seq was first established using two distinct cell lines; normal pancreatic duct cells (hTERT-HPNE) and pancreatic ductal adenocarcinoma (PDAC) cells (Panc04.03). The two cell lines were accurately classified based on mRNA and glycan alone, as well as by the integration of the two omics data (total of 11,907 cells). Distinct differences in the lectin binding intensity indicating different glycan profiles were observed between the two cell lines. The lectin binding profiles obtained from scGR-seq were validated by flow cytometry analysis using fluorescent-labeled lectins which showed consistent results indicating the reliability of droplet-based scGR-seq data [27].

**Fig. 2:**
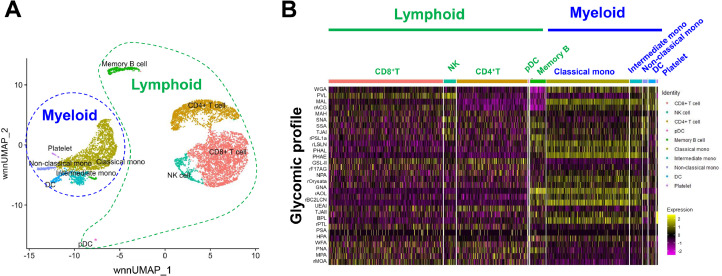
Application of droplet-based scGR-seq for the glycomic and transcriptomic profiling of PBMC. A) Clustering of the cell types based on the integrated information of glycan and mRNA by UMAP. B) Heatmap showing lectin binding intensities to each cell types of PBMC. *Yellow* to *pink* color gradient change represents high to low lectin binding intensity. This figure was modified from the previous report [25].

**Fig. 3:**
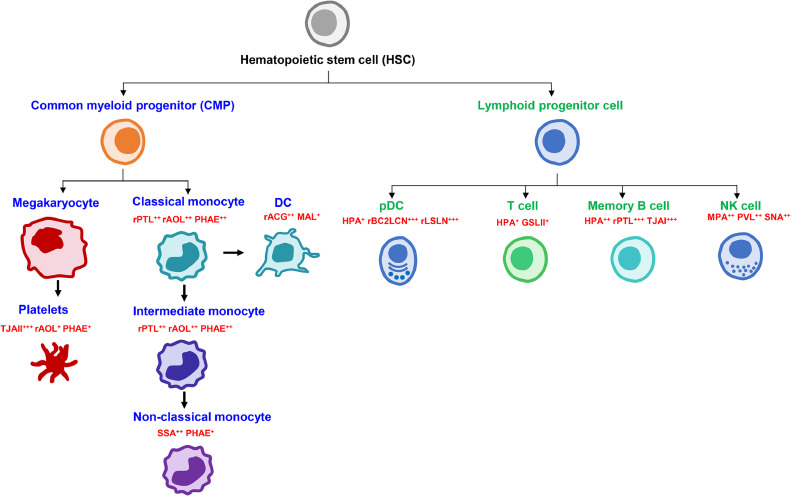
Glycan signature of each cell type obtained from lectin reactivity. The averaged lectin intensity ranges from > 2 to < −2 (+++ ≥ 2: very high, ++ ≥ 1: high, + > 0: mid). This figure was modified from the previous report [25].

The application of droplet-based scGR-seq was challenged to a more complex sample; peripheral blood mononuclear cells (PBMC), consisting of various immune cell types. Non-linear clustering of cells by UMAP, based on the integrated mRNA and glycan information identified a rare cell population “pDCs (plasmacytoid dendritic cells)”, present in a very small amount in the blood, in addition to the cell types identified using mRNA information alone (total 8000 cells). The cells were largely grouped into two main clusters based on the lineage: myeloid (classical monocytes, intermediate monocytes, non-classical monocytes, platelets, dendritic cells (DCs) and lymphoid (CD4^+^ T cells, CD8^+^ T cells, NK cells, memory B cells, pDCs) (Fig. 2A). The lectin binding intensity and the patterns differed across cell types, as shown in the heatmap in Fig. 2B. Variations in the lectin binding intensities were also observed within the same cell type, highlighting the heterogeneity of glycans. The α1–2Fuc-binding lectin, TJAII, was identified as a lectin probe that reacts substantially higher to platelets compared to other cell types, which were also validated by flow cytometry analysis. Similar to the PLS regression analysis performed on plate-based scGR-seq data, we could also analyze the association of glycan and mRNA expression by PLS regression. From PLS analysis, the novel association of TJAII with gene group involved in biological function for platelets such as “blood coagulation” and “regulation of platelet activation” was observed. The glycoprotein ligand for TJAII was identified as the platelet glycoprotein GPIb-α, whose interaction with von Willebrand factor (vWF) increases after the removal of fucose, suggesting an important role of α1–2Fuc on platelet in blood coagulation. Although TJAII reacts strongly to platelets, α1–2 fucosyltransferase genes (*FUT1 and FUT2*) expression, which are responsible for synthesizing α1–2 fucose, could not be detected due to the inherently low-level expression of glycosyltransferase genes. Such discrepancy between the glycans identified by the lectin probe in both scGR-seq and flow cytometry, and the expression of glycosyltransferase genes, were frequently observed, indicating the complex factors influencing glycosylation. These findings also highlight the advantage of droplet-based scGR-seq in accurately detecting cell surface glycans where the glyco-genome expression is not accessible. From the lectin reactivity obtained through droplet-based scGR-seq data, the glycan signature for each cell type within PBMC could be defined at once [27] (Fig. 3).

## 6 Other related methods

Regarding single-cell glycome profiling technology, several other technologies have been reported such as SUrface-protein Glycan And RNA-seq (SUGAR-seq), CyTOF-Lec, CE-MS, Glycopacity, and deep learning methods [[28], [29], [30], [31], [32]].

Around the same time as the first publication of scGR-seq [23], SUGAR-seq was reported [28], which combines one lectin with multiple antibodies for single-cell analysis on the 10x Genomics droplet-based analysis platform. PHA-L, a lectin specific for complex-type N-glycans, is biotinylated to form a complex with DNA barcode-conjugated streptavidin to enable sequencing. The authors analyzed tumor-infiltrating T cells with this technology, demonstrating the classification of tumor-infiltrating T cell subpopulations by multi-branched N-glycans. On the other hand, this technique utilized only one type of lectin, limiting the ability to analyze the entire glycan profile.

As a different approach from sequencing using DNA barcodes, a paper on single-cell glycan profiling using Cytometry by time-of-flight (CyTOF) was reported in 2022 [29]. CyTOF employs mass spectrometry to measure metal isotope-labeled antibodies bound to single cells, enabling multiplex analysis of numerous parameters simultaneously. In this paper, the reactivity of five lectins (two sialic acid-binding lectins, two fucose-binding lectins, and one T antigen-binding lectin) and 34 antibodies were simultaneously analyzed by CyTOF, which is called the CyTOF-Lec method. This method was used to analyze HIV virus-infected cells, demonstrating enhanced fucosylation and sialylation of glycans in HIV infection, and memory CD4^+^ T cells with high levels of sialylated glycans are susceptible to HIV infection. On the other hand, this method does not provide gene expression information, making it impossible to analyze the relationship between detailed cell type/cell state and glycan expression.

Another paper using CE-MS (capillary electrophoresis coupled with mass spectrometry) for single-cell glycan analysis was reported in 2024 [30]. In this method, N-glycans are released from a small number of blood-derived isolates as well as from cell surfaces within the capillary coupled with label-free high-sensitivity CE-MS analysis for direct detection and structural analysis of native N-glycans in single-cell. Relative abundance of heavily fucosylated and sialylated N-glycans could be detected from a single cell using this method. The method was able to detect glycan heterogeneity within the same cell type. This method was standardized for the analysis of a small number of samples which could be useful for glycan detection in limited samples. However, the current method focuses solely on N-glycan, which limits the overall understanding of the glycomic landscape of the cells. Additionally, the technique does not allow for the integrated omic analysis, although the author stated the prospect of additional omic approaches [30].

Bioinformatic approaches utilizing publicly available scRNA-seq data to predict glycan profiles have also been reported. In 2022, a study reported the development of a software called Glycopacity. Glycogenes with distinct expression patterns across organs and cell types are defined as hotspots that shape cell-specific glycan profiles. This approach was adapted to a scRNA-seq public data set to predict the glycan structure characteristics of each cell by mapping them onto known glycan synthesis pathways [31]. On the other hand, as discussed by the authors of this paper, it is difficult to obtain reliable data with scRNA-seq because of the inherently low expression levels of glycosyltransferase genes. The analysis requires integrating data from over 200 cells into PseudoBulk, making rare cell clusters difficult to study. In addition, since glycans are synthesized by various gene products, it is difficult to accurately predict glycan structures from gene expression data alone.

Another paper published in 2022 incorporates a deep learning (DL) model on SUGAR-seq data to predict glycosylation phenotype [32]. In this study, by training a neural network model using multi-omics data (scRNA-seq and lectin-seq) from mouse T-cells, the presence of β1–6GlcNAc branching across T-cell subtypes was predicted with high precision. Low-abundance critical genes involved in glycan regulation, that impact branched glycan levels and their biological roles, the expression of which are not detected by conventional differential expression analysis, were identified by DL model interpretation. This study highlights the potential of deep learning in multi-omics data to uncover novel functions and regulatory mechanisms of glycans.

## 7 Concluding remarks and future perspective

The scGR-seq technology enables multi-modal analysis of glycome and transcriptome in thousands of single cells at once. Glycome data for each cell type and each cell state within a mixed cell population can be obtained in a single experiment without prior purification of each cell type or subpopulation. Thus, droplet-based scGR-seq is particularly suitable for the analysis of complex samples such as tumor tissues, for the discovery of the glycan marker and expression in the tumor microenvironment. Additionally, it can capture the glycome information of rare cells, such as circulating tumor cells in the blood, which can be applied for drug target discovery. Since the current method uses lectins for glycan profiling, the glycans identified are limited by the specificity of the lectins used, the glycan amount cannot be measured, and the precise glycan structure cannot be fully determined. By further incorporating deep learning models into the dataset generated from scGR-seq, novel functions and regulatory mechanisms of glycans can be elucidated. Overall, the scGR-seq technology represents a powerful technology that can greatly advance our understanding of single-cell glycomics.

## Funding

This work was supported by JSPS KAKEN (23K26872, 23H04796), JST A-step (JPMJTR23U6), and AMED e-Asia JRP (23jm0210100h0002).

## CRediT authorship contribution statement

**Sunada Keisham:** Writing – review & editing, Writing – original draft, Formal analysis, Data curation. **Hiroaki Tateno:** Writing – review & editing, Writing – original draft, Methodology, Funding acquisition, Data curation, Conceptualization.

## Declaration of competing interest

The authors declare that they have no known competing financial interests or personal relationships that could have appeared to influence the work reported in this paper.

## Data Availability

No data was used for the research described in the article.
